# Effects of Spine Motion on Foot Slip in Quadruped Bounding

**DOI:** 10.1155/2018/8097371

**Published:** 2018-03-25

**Authors:** Dongliang Chen, Ningjie Li, Guifang Liu, Lei Chen, Yongyuan Wang, Chong Liu, Bo Zhuang

**Affiliations:** College of Mechanical and Electrical Engineering, Harbin Engineering University, Harbin 150001, China

## Abstract

Translation and bend of the spine in the sagittal plane during high-speed quadruped running were investigated. The effect of the two spine motions on slip between the foot and the ground was also explored. First, three simplified sagittal plane models of quadruped mammals were studied in symmetric bounding. The first model's trunk allowed no relative motion, the second model allowed only trunk bend, and the third model allowed both bend and translation. Next, torque was introduced to equivalently replace spine motion and the possibility of foot slip of the three models was analyzed theoretically. The results indicate that the third model has the least possibility of slip. This conclusion was further confirmed by simulation experiments. Finally, the conclusion was verified by the reductive model crawling robot.

## 1. Introduction

Spine motion is usually visually apparent in quadruped mammals (especially the cheetah) during high-speed running. Many researchers believe that quadruped mammals use their spines to increase stride length [1], supply extra power [2], enhance stability [3], and improve energy efficiency [4]. Inspired by these observations, many robotics researchers have taken a strong interest in the quadruped robot with flexible spines. In a recent study, Khoramshahi et al. [5] researched linear spine for speed-energy efficiency trade-off in quadruped robots. Kani et al. [6, 7] explored the effect of flexible spine on stability and bounding gait of a passive quadruped robot. Cao and Poulakakis, Eckert et al., and Çulha and Saranli [8–10] focused on developing effective control methods to achieve high-speed gaits with flexible spines. However, the effect of spine motion on foot slip has not been studied systematically.

Generally, bending is the only spine motion considered in the sagittal plane. Zhao and Chen et al. [11, 12] simplified the sagittal plane model of quadruped mammals. The trunk is articulated in the sagittal plane model, and it can be termed an articulated spine model (ASM). But the performance of an ASM is greatly inferior to that of a real animal in terms of locomotion speed, stability, and energy efficiency [13, 14]. Therefore, bend may be only one main form of spine motion, and others may be also important.

A real animals' spine consists of many vertebrae with soft spaces between them filled by ligaments and intervertebral discs. The ligaments and intervertebral discs act as shock absorbers [15]. So translation between vertebrae is another spine motion in the sagittal plane which may be important during high-speed quadruped running.

In fact, there is another simplified sagittal plane model of quadruped mammals, where the trunk travels with an inchworm-like motion (translation) [16, 17]. However, this model has an obvious deficiency that parts of the trunk cannot rotate with respect to one another. Hence, translation and bend should be considered simultaneously in the study of spine motion.

This research investigated two spine motions—translation and bend—during quadruped running using three simplified sagittal plane models of quadruped mammals. Furthermore, the effect of the two spine motions on foot slip was assessed.

## 2. Models

### 2.1. Model Description

**Figure 1:**
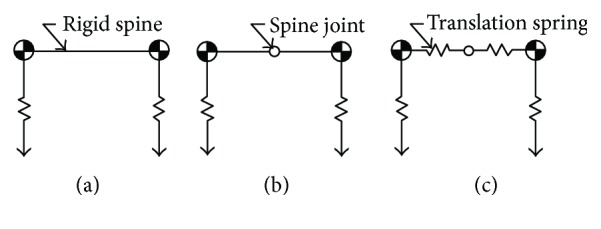
Three simplified sagittal plane models of quadruped mammals: (a) RSM; (b) ASM; (c) TASM.

Three simplified sagittal plane models of quadruped mammals are depicted in Figure 1. A rigid spine model (RSM) is shown in Figure 1(a). The mass of the rigid beam is represented as a point mass, and the two rigid beams are connected by a lock structure. The leg systems are represented as massless springs. As both the front and rear legs are in phase during bounding, only one spring leg is attached to each end of the trunk (front and rear).

Figure 1(b) depicts an ASM. The two rigid beams are connected by an articulated joint. The spine motion is actuated. An ASM is the same as an RSM except that there is no spine joint in the trunk. The ASM and RSM are set as two baseline models in this study.

The key objective model of this study is shown in Figure 1(c). We get this model inspired by the vertebrae of the real spine which leads to a least likelihood of sliding [18, 19]. We describe this as a translational and articulated spine model (TASM). The trunk includes a spine joint and two springs with a single degree of freedom, all connected in series. The two springs can only be compressed. The other parts of the TASM are all the same as those of the ASM.

### 2.2. Spine Motions

**Figure 2:**
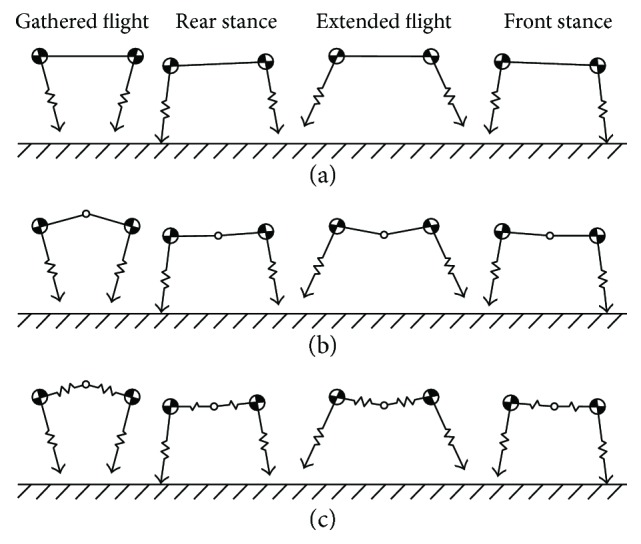
Spine motions in the four phases of symmetric bounding as represented in the (a) RSM, (b) ASM, and (c) TASM.

Hoyt and Taylor [20] showed that an energy minimum exists at an animal's naturally selected gait within the speed range. For high-speed running, the rotary gallop is the chosen gait. A detailed description of the rotary gallop has been provided by Hildebrand [21]. By pairing both the fore and hind legs, the rotary gallop can be simplified to a symmetric bounding gait. The symmetric bounding gait is very suitable for simplified sagittal plane models of quadruped mammals [22–24]. A full symmetric bounding cycle is usually deconstructed into four typical phases: the gathered flight phase, the rear stance phase, the extended flight phase, and the front stance phase. The spine motions in the four phases as represented in the RSM, ASM, and TASM are shown in Figures 2(a), 2(b), and 2(c), respectively.

In Figure 2(a), the robot has no spine motion in a full symmetric bounding cycle. The flight phases (the gathered flight phase and the extended flight phase) of Figure 2(b) and Figure 2(c) have the same spine motion. In the stance phases (the front leg stance phase and the rear leg stance phase) of Figure 2(b), only bending motion exists in the trunk. However, in the stance phases of Figure 2(c), both bending and translation motions are allowed in the trunk.

## 3. The Theoretical Analysis of Foot Slip

The hypothetical research environment is flat. Slip between the foot and the ground is a common instability when a quadruped mammal runs on flat ground. Therefore, analyzing the possibility of foot slip of the three models (the RSM, ASM, and TASM) can assess which model is better in modeling a real animals' spine motion in the sagittal plane.

**Figure 3:**
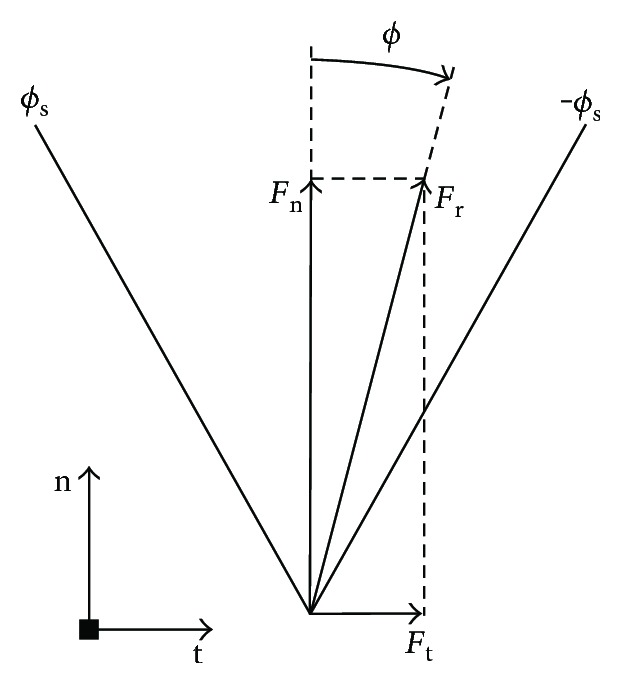
Static friction cone.

In many studies of quadruped robots with flexible spines, the foot is assumed “locked” to the ground at the contact points [25, 26]. In this study it is not, because the interaction forces between the foot and the ground must be studied. As the three models are all planar, the interaction resultant is also located in the sagittal plane. Based on the theorem of Coulomb friction, there is a no-slip constraint known as the static friction cone. The static friction cone is depicted in Figure 3, and the relationships involved can be expressed as

(1) $$\begin{array}{c} \phi =\arctan\left(-\frac{F_{t}}{F_{n}}\right),\\ \phi_{s}=\arctan\left(u_{s}\right),\\ -\phi_{s}<\phi <\phi_{s},\; \end{array}$$

where *u*_s_ is the static friction coefficient between the foot and the ground, *ϕ*_s_ is the angle of static friction, *F*_r_ is the interaction resultant, *F*_n_ is the normal component of *F*_r_, *F*_t_ is the horizontal component of *F*_r_, and *ϕ* is the angle from the vertical axis to the action line of *F*_r_. The value of *ϕ* is defined as positive in the counterclockwise direction and negative in the clockwise direction.

From (1), the main factor determining slip is the value of *ϕ*. To simplify the analysis of that main factor, torque *τ*_s_ was introduced to equivalently replace spine motion. So we have

(2) $$\begin{array}{c} \tau_{s}=\tau_{r}+\tau_{t}, \end{array}$$

where *τ*_r_ is the torque equivalently replacing trunk bend and *τ*_t_ is the torque equivalently replacing trunk translation. The values of *τ*_s_, *τ*_r_, and *τ*_t_ are defined as positive in the counterclockwise direction and negative in the clockwise direction.

**Figure 4:**
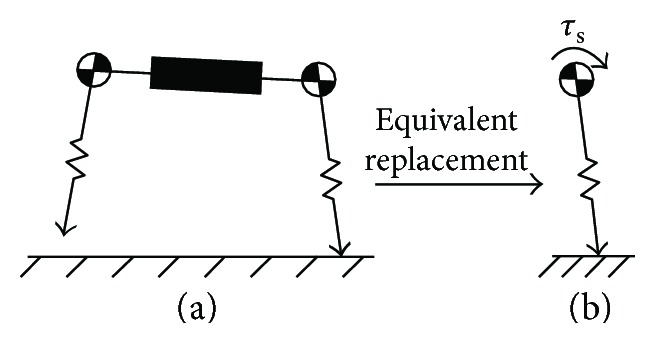
Equivalent replacement of spine motion: (a) RSM, ASM, or TASM in the front leg stance phase; (b) equivalent model.

**Figure 5:**
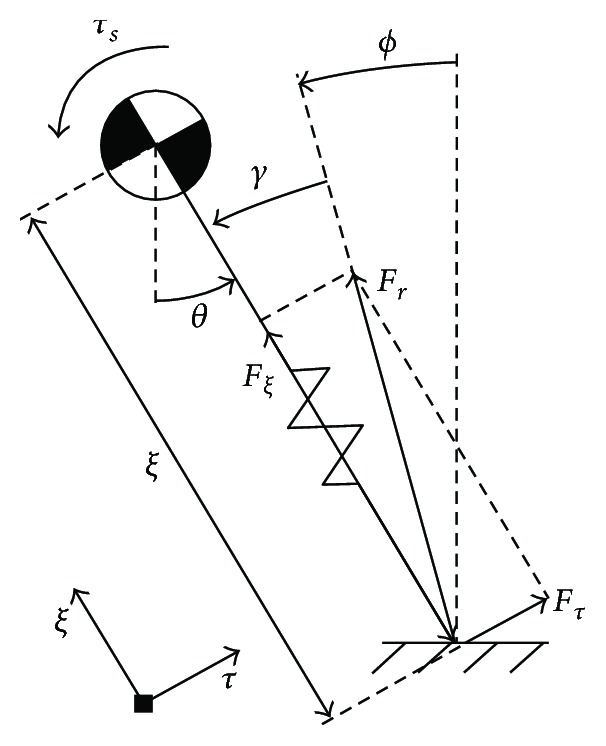
Free-body diagram of the EM.

Figure 4 shows how to define an equivalent replacement. Figure 4(a) shows the RSM, ASM, or TASM in the front leg stance phase, where the black box stands for the different trunks of the three models. Figure 4(b) depicts the equivalent model (EM) where *τ*_s_ is applied about the position of the point mass. Its free-body diagram is shown in Figure 5, where *F*_r_ is decomposed under a local rectangular coordinate system. Based on Figure 5, before slip occurs, there is an equation:

(3) $$\begin{array}{c} F_{\tau }\xi +\tau_{s}=0,\\ \gamma =\arctan\left(\frac{F_{\tau }}{F_{\xi }}\right),\\ \theta =\gamma +\phi , \end{array}$$

where *ξ* is the length of the standing leg, *F_ξ_* is the component of *F*_r_ parallel to the center line of the standing leg, *F_τ_* is the component of *F*_r_ perpendicular to the center line of the standing leg, *γ* is the angle from the action line of *F*_r_ to the center line of the standing leg, and *θ* is the angle from the vertical axis to the center line of the standing leg. The values of *γ* and *θ* are defined as positive in the counterclockwise direction and negative in the clockwise direction.

From (3), *ϕ* can be given by

(4) $$\begin{array}{c} \phi =\theta -\arctan\left(-\frac{\tau_{s}}{\xi F_{\xi }}\right). \end{array}$$

When the RSM, ASM, or TASM is in the rear leg stance phase, (4) is still appropriate. From (1) and (4), before foot slip occurs, the range of the value of *θ* can be used to quantify the possibility of foot slip. The larger (smaller) the range of the value of *θ*, the lower (higher) is the possibility of foot slip. The value of *θ* can be calculated using (4). *θ*(*t*_1_) is the value of *θ* at the time of *ϕ* = *ϕ*_s_ and *θ*(*t*_2_) is its value when *ϕ* = −*ϕ*_s_. Combined with a constraint that the range of *θ* values is symmetric in a full symmetric bounding cycle, the maximum value of *θ* can be given by

(5) $$\begin{array}{c} \theta_{\max}=\min\left(\theta \left(t_{1}\right),\left|\theta \left(t_{2}\right)\right|\right), \end{array}$$

where *θ*_max_ is the maximum value of *θ*. Because spine motions of the RSM, ASM, and TASM are different, it is necessary to discuss *θ*_max_ with the three models one by one.

In the RSM, there is no spine motion in the stance phases of a full symmetric bounding cycle. So for the RSM, (2) can be rewritten as

(6) $$\begin{array}{c} \tau_{s}=0. \end{array}$$

With (4), (5), and (6), *θ*_max_ in the RSM can be given by

(7) $$\begin{array}{c} \theta_{\max}=\phi_{s}. \end{array}$$

For the ASM, only the trunk is bending in the stance phases of a bounding cycle. So for the ASM, (2) can be rewritten as

(8) $$\begin{array}{c} \tau_{s}=\tau_{r}. \end{array}$$

When *ϕ* = *ϕ*_s_ or *ϕ* = −*ϕ*_s_, the values of *ξ* and *F_ξ_* are positive and the value of *τ*_r_ is negative, which can be got from Figures 4 and 5. Combined with (4), (5), and (8), *θ*_max_ in the ASM can be given by

(9) $$\begin{array}{c} \theta_{\max}=\phi_{s}-\sigma_{1}, \end{array}$$

where *σ*_1_ is a positive number.

The TASM allows both trunk bending and translation in the stance phases. From Figures 4 and 5, we can get the following: at the time of *ϕ* = *ϕ*_s_, *ξ* and *F_ξ_* are both positive and *τ*_r_ and *τ*_t_ are both negative; when *ϕ* = −*ϕ*_s_, *ξ*, *F_ξ_*, and *τ*_t_ are all positive and *τ*_r_ is negative. Under the condition

(10) $$\begin{array}{c} \tau_{r}\left(t_{2}\right)+\tau_{t}\left(t_{2}\right)>0, \end{array}$$

and combining with (2), (4), and (5), *θ*_max_ in the TASM can be given by

(11) $$\begin{array}{c} \theta_{\max}=\phi_{s}+\sigma_{2}, \end{array}$$

where *σ*_2_ is another positive number.

Comparing (7), (9), and (11), it is obvious that the TASM has the largest range of *θ* values, which means it has the least possibility of foot slip. Therefore, the TASM is the best among the three models in modeling a real animals' spine motion in the sagittal plane. Although *θ*_max_ in the TASM (11) is got under (10), (10) is only possible in the TASM among the three models.

## 4. Simulation Experiments

### 4.1. Simulation Environment

**Figure 6:**
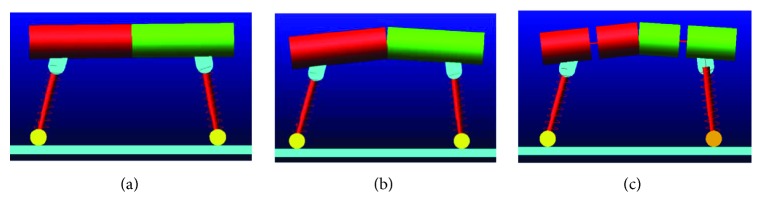
Three simulation models: (a) RSM; (b) ASM; (c) TASM.

**Figure 7:**
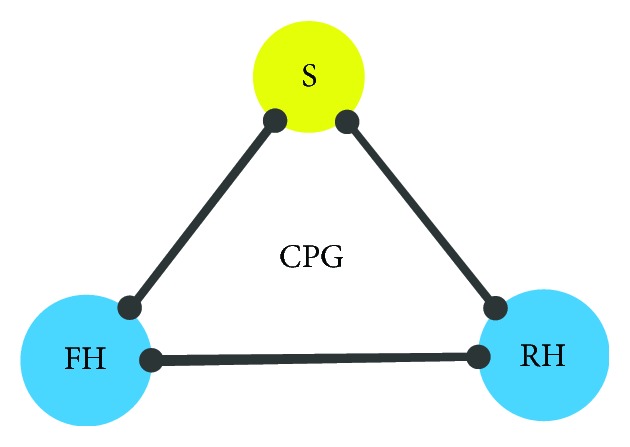
Structure of the model's controller.

The simulation experiments were carried out to confirm the results of the theoretical analysis. The simulation models are shown in Figure 6. The EM was chosen as the simulation analysis model, as shown in Figure 4(b). Raibert's “three-part” control method [23, 27] was applied to the EM, for gaining its bounding cycles. The structure of the model's controller is shown in Figure 7. In Figure 7, the labels indicated are as follows: S for spineoscillator, FH for front hip joint oscillator, and RH for rear hip joint oscillator. The controller includes three oscillators. Each oscillator controls one active joint, the spine joint, and the two hip joints. Note that the oscillator is the basic component of CPG. The mathematical expression of the oscillator can be given by

(12) $$\begin{array}{c} {\dot{\phi }}_{i}=2\pi f+\sum_{j\neq i}k_{\left(i,j\right)}\sin\left(\phi_{j}-\phi_{i}-\phi_{\left(i,j\right)}\right),\\ a_{i}=\alpha \left(A_{i}-a_{i}\right),\\ o_{i}=\alpha \left(O_{i}-o_{i}\right),\\ \theta_{i}=\left\{\begin{array}{ll} \frac{\phi_{i}}{2D_{vir}}, & 0\leq \phi_{i}\leq 2\pi D_{vir},\\ \frac{\phi_{i}+2\pi \left(1-2D_{vir}\right)}{2\left(1-2D_{vir}\right)}, & 0\geq 2\pi D_{vir}, \end{array}\right.\\ \Gamma_{i}=a_{i}\cos\left(\theta_{i}\right)+o_{i}, \end{array}$$

where *i* and *j* represent the numbering of CPG three oscillators; S, FH, and RH are 1, 2, and 3, respectively. The parameters of the motion controller are defined in Table 1.

In the theoretical analysis, spine motions of the RSM, ASM, and TASM were equivalently replaced by torque *τ*_s_; the RSM, ASM, and TASM were all simplified as the EM; and the characteristics of torque *τ*_s_ of the RSM, ASM, and TASM were discussed one by one ((6), (8), and (2)). The simulation environments were MSC Adams and Matlab-Simulink. MSC Adams was used to calculate dynamic equations of the EM and record its performance. Matlab-Simulink was used to run the “three-part” controller. In MSC Adams, the contact mechanic between the foot and the ground was set based on the theorem of Coulomb for friction, as in the theoretical analysis. The parameter static friction coefficient *u*_s_ was set as 0.4; *ϕ*_s_ was 21.8°, calculated based on (1). The communication interval between MSC Adams and Matlab-Simulink was set as 0.002 second.

The overall parameters of the simulation model we designed are shown in Table 2. The front and rear body parts of the robot are replaced by cylinders, and the legs are replaced by lightweight springs. Because of the leg stiffness, the stiffness of the spine, and the angle of the ground, these have a great effect on the experimental results, so these factors are selected by experiment. The following will be a detailed introduction. In order to evaluate the results of the simulation experiments, we provided a set of performance metrics including stability behavior and cost of transport.

#### 4.1.1. Leg Stiffness.

**Figure 8:**
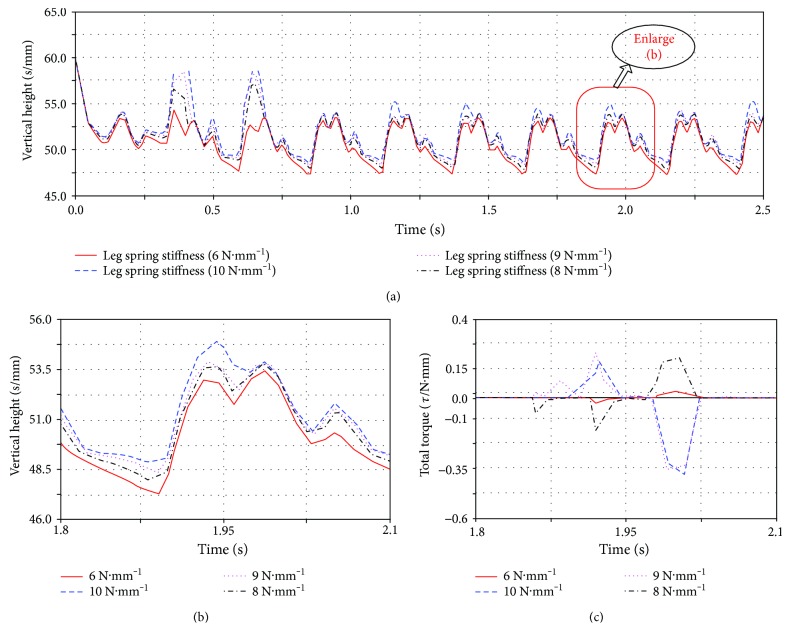
Effect of leg stiffness on simulation model: (a) the vertical height of the model centroid; (b) the centroid of a cycle of displacement; (c) the total torque required to simulate the model.

The effect of the legs on the robot is very large. The legs are simplified to spring systems according to the bionic principle. This will be closer to the really quadruped mammals. Different leg stiffness produce different experimental results. The optimal leg stiffness is selected by simulation experiments to minimize the effect of the leg on the experiment. The simulation results are shown in Figures 8(a)–8(c). When the stiffness is 8 N·mm^−1^, the comprehensive performance is the best in Figure 8. Note that because we have a lot of simulation cycles (Figure 8(a)), in order to facilitate comparison, we intercept one of the stable cycles to compare (Figure 8(b)). The latter two sets of data are also cut off a cycle.

#### 4.1.2. Stiffness of the Spine.

**Figure 9:**
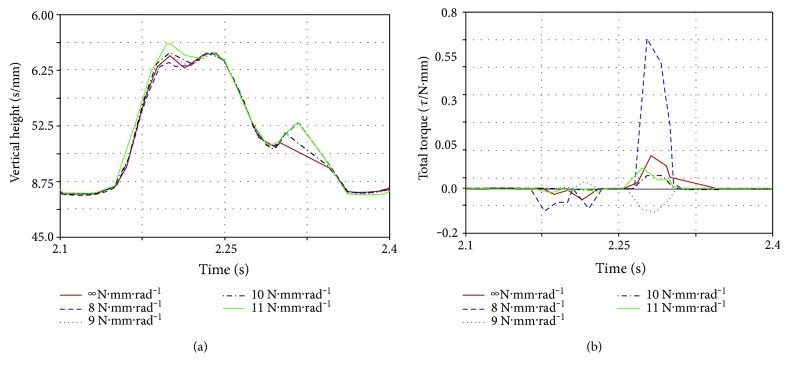
Effect of spine stiffness on simulation model: (a) simulated location of the model particle; (b) the total torque required to simulate the model.

The spine simulation experiment uses a motor with a torsion spring. The motor provides the driving power of the spine, and the torsion spring can increase the flexibility of the spine. The stiffness of different torsion spring will have different effects. Experiments were carried out on the stiffness of different torsion springs. The simulation results are shown in Figures 9(a) and 9(b). When the stiffness is 10 N·mm·rad^−1^, the comprehensive performance is the best.

#### 4.1.3. Grounding Angle.

**Figure 10:**
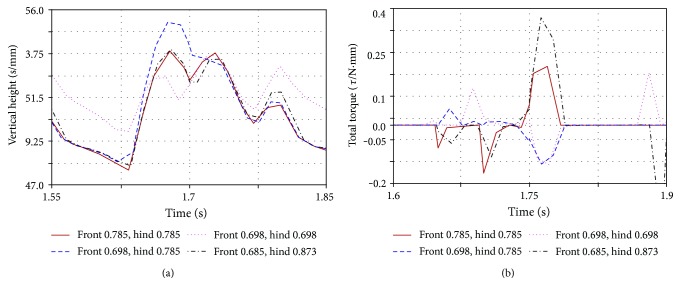
Effect of grounding angle on simulation model: (a) simulated location of the model particle; (b) the total torque required to simulate the model.

The ground angle determines the attitude of the robot's leg. The size of the grounding angle will have a great impact on the motion of the robot. So the best grounding angle are chosen to complete simulation experiments. The final choice of the front leg grounding angle is 0.698 rad and the hind leg grounding angle is 0.785 rad (Figures 10(a) and 10(b)).

### 4.2. Simulation Experiments

Three simulation experiments were done. At the beginning of each simulation experiment, the desired forward speed of the simulation model was specified as 3500 mm·s^−1^. Then, the simulation model bounded and accelerated. If foot slip occurred, the simulation experiment ended at the time of foot slip occurring. If foot slip did not occur, the simulation experiment ended when the simulation models bounded stably at the desired forward speed. In each simulation experiment, performance indicators of the EM, such as *ν* (forward speed), *θ*, and *ϕ*, were recorded from the start to the end.

**Figure 11:**
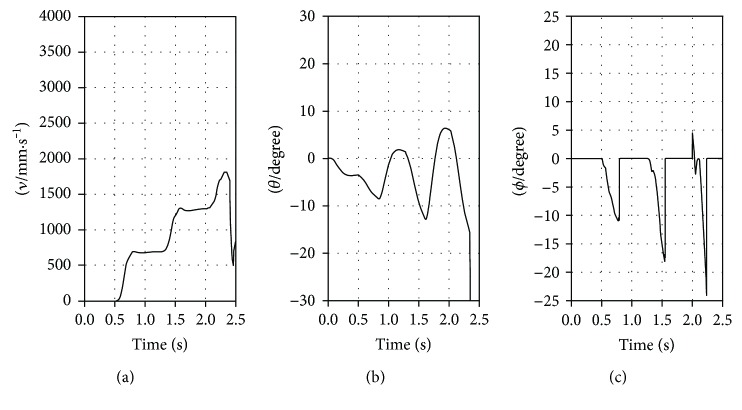
Results of the simulation experiment representing the RSM: (a) *ν*-time; (b) *θ*-time; (c) *ϕ*-time.

**Figure 12:**
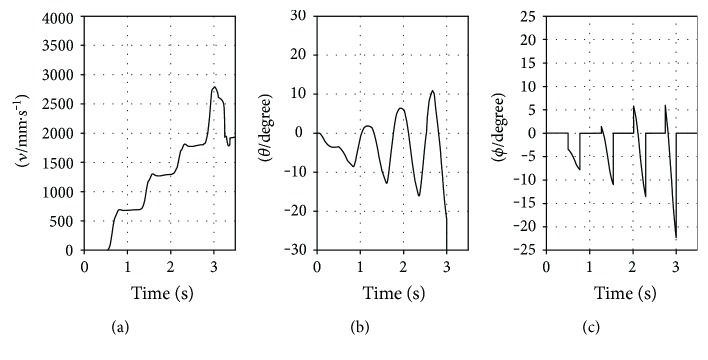
Results of the simulation experiment representing the ASM: (a) *ν*-time; (b) *θ*-time; (c) *ϕ*-time.

**Figure 13:**
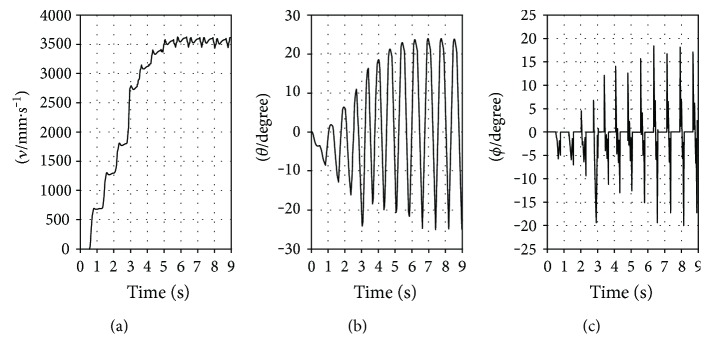
Results of the simulation experiment representing the TASM: (a) *ν*-time; (b) *θ*-time; (c) *ϕ*-time.

The results of the three simulation experiments are plotted in Figures 11, 12, and 13 for the RSM, ASM, and TASM, respectively. The RSM slipped when the value of *ν* increased to about 1800 mm·s^−1^ (Figure 11(a)). The value of *ϕ* was then about −24° (Figure 11(c)), which is beyond −*ϕ*_s_ (−21.8°). The ASM also slipped when the value of *ν* increased to about 2700 mm·s^−1^ (Figure 12(a)). The value of *ϕ* was about −23° at that point (Figure 12(c)), which is also beyond −*ϕ*_s_. However, the TASM did not slip and bounded stably at the desired forward speed of 3500 mm·s^−1^ (Figure 13(a)). The value of *ϕ* in the TASM was always within the static friction cone (Figure 13(c)). Before slip occurred, the maximum value of *θ* for the RSM and ASM was about 11° (Figure 11(b)) and 7° (Figure 12(b)), respectively. For the TASM, the maximum value of *θ* was 24° (Figure 13(b)). Clearly, the range of *θ* values in the TASM is the largest and the TASM has the least possibility of slipping. And the TASM can bound at a faster speed than the RSM and ASM.

## 5. Crawling Experiments

### 5.1. Experiment Environment

In order to verify the correctness of the theory, we have done a simple experimental model. The translational spine motion of the robot can be abstractly turned into the motion of the car. The bending motion of the spine can improve the athletic performance of the quadruped robot, which has been studied by our laboratory [12]. In order to express the translational spine motion of the spine more clearly, we designed this experiment.

**Figure 14:**
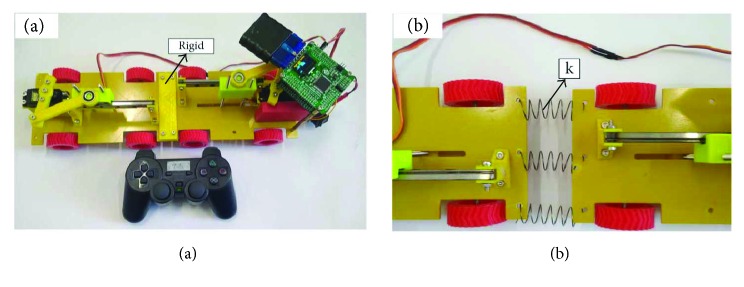
Crawling robot: (a) crawling robot with rigid spine; (b) crawling robot with linear spring (simulate the translation of the spine).

The robot has two parts before and after the middle of the spring connection. The slider is used for the robot as a power source. The front body has an actuation slider and the rear body also has one. In order to ensure the linear motion of the slider, a linear slide is added to restrain the motion. The slider is controlled by a servo motor with a sinusoidal signal. The slider is in contact with the ground and produces forward and backward forces. The slider is wedged with the ground. The robot crawls forward because the forward friction is less than the backward friction. We use the battery to provide power, and the controller can provide location information. Each body has four passive friction wheels. For rigid spine motion, we connect the front body and the rear body with a rigid plate, as shown in Figure 14(a). For the translation motion of the spine, we use the spring to connect the body (Figure 14(b)). Nevertheless, there are several uncertain factors in the test, such as the shape of the slider, the rubbing in both directions, the accuracy of the sensor and the controller.

### 5.2. Crawling Experiments

In the experiment, in order to ensure the accuracy of the results, we set a single variable. The experiment keeps the voltage constant. The robot crawls the same distance, measuring the time required for different spine motions. By measuring the position of the two sliders, the phase difference of the controller is determined. After several experiments, we found that when the phase difference between the two controllers is 180 degrees, the robot's motion performance is the best. When the phase difference is determined, the current is constant when the voltage is the same. The time spent in each experiment was recorded to compare the motion of the spine. Three repetitive experiments were done for each spinal motion.

In all experiments, the linear spring was significantly better than the rigid case, as shown in Table 3. In the first group, the spine of the linear spring was 8.05% better than the rigid (compare case 1 and case 4). The other three groups correspond to 6.05%, 1.20%, and −1.83%, respectively. This result confirms our expectations and simulation results. That is to say, the translational spine motion of the robot can effectively reduce the foot slip. When the linear spring stiffness is very small, it will lose the superiority of the motion. This is because the spring stiffness is relatively small; it will slow down the process of the spring storage and release of the energy. This can not reach the needs of robot motion. The original behavior of the robot motion will be reduced.

## 6. Conclusions

In this paper, we have made a systematic study of the proposed spinal motion, including bending and translational motion. This spine motion has a beneficial effect on quadruped robots. The TASM has demonstrated to have the least possibility of foot slip among the three simplified sagittal plane models of quadruped mammals. Trunk translation and bending represented in the same model can enhance the system's stability. Therefore, in order to model a real animals' spine motion effectively, trunk translation and bending should be used together. This finding is helpful for robotics researchers to get better understanding of real animals' spine motion. The trunk structure of the TASM is also a good suggestion for further study of quadruped robots with flexible spines.

## Figures and Tables

**Table 1:** Parameters of the motion controller.

Parameter	Definition
*f*	Gait frequency
*ϕ*_*i*_	Oscillator phase
*A*	Joint motion ideal amplitude
*O*	Ideal offset of joint motion
*θ*	Joint motion phase
*k*, *α*	Proportional control factor
*D*_vir_	Gait ideal load factor
*a*	Instantaneous amplitude
*o*	Instantaneous offset
*Γ*	Joint motion position

**Table 2:** Parameters of the simulation model.

Parameter	Value
Half body length (*l*, mm)	60
Half body mass (*m*, kg)	1
Leg free length (*l*_0_, mm)	50
Leg spring stiffness (*k*, N·mm^−1^)	8
Spine stiffness of the simulation model (*k*_r_, N·mm·rad^−1^)	10
The simulation of the translation of the spine stiffness (*k*_t_, N·mm·rad^−1^)	9
Front leg ground angle (rad)	0.698
Hind leg ground angle (rad)	0.785

**Table 3:** Result for crawling robot.

Case	Spring type	*K* (N·mm^−1^)	Crawling time (s)	Performance improvement over the rigid case
1	Linear spring, normal	7.54	16.11	8.05%
2	Linear spring, hard	16.42	16.46	6.05%
3	Linear spring, soft	3.60	17.31	1.20%
4	Rigid	∞	17.52	0.00%
5	Linear spring, very soft	0.51	17.84	−1.83%

The spine stiffness of crawling robot with rigid spine is ∞.
